# Male Gender, Increased Blood Viscosity, Body Mass Index and Triglyceride Levels Are Independently Associated with Systemic Relative Hypertension in Sickle Cell Anemia

**DOI:** 10.1371/journal.pone.0066004

**Published:** 2013-06-13

**Authors:** Yann Lamarre, Marie-Laure Lalanne-Mistrih, Marc Romana, Nathalie Lemonne, Daniele Mougenel, Xavier Waltz, Benoît Tressières, Maryse Etienne-Julan, Vanessa Tarer, Marie-Dominique Hardy-Dessources, Philippe Connes

**Affiliations:** 1 Institut National de la Santé et de la Recherche Médicale UMR 665, Hôpital Ricou, Centre Hospitalier Universitaire de Pointe-à-Pitre, Pointe-à-Pitre, Guadeloupe; 2 Université des Antilles et de la Guyane, Pointe-à-Pitre, Guadeloupe; 3 Laboratoire d’excellence GR-Ex «The red cell: from genesis to death», PRES Sorbonne Paris Cité, Paris, France; 4 Centre d’Iinvestigation Clinique - Epidémiologie Clinique 802 Inserm Antilles-Guyane, Centre Hospitalier Universitaire de Pointe-à-Pitre, Pointe-à-Pitre, Guadeloupe; 5 Unité Transversale de la Drépanocytose, Centre Hospitalier Universitaire de Pointe-à-Pitre, Pointe-à-Pitre, Guadeloupe; 6 Laboratoire EA3596, Département de Physiologie, Université des Antilles et de la Guyane, Pointe-à-Pitre, Guadeloupe; Emory University/Georgia Insititute of Technology, United States of America

## Abstract

Patients with sickle cell anemia (SCA) have usually lower diastolic, systolic and mean blood pressure (BP) than the general population. However, BP values ≥120/70 mmHg considerably increase the risk for acute and chronic complications in SCA. The aim of this study was to identify biological factors associated with relative hypertension in adults with SCA. We compared the hematological, lipid and hemolytic profiles, as well as blood viscosity, between SCA patients with normal BP (<120/70 mmHg, n = 54) and those with relative hypertension (BP≥120/70 mmHg, n = 43). Our results demonstrated that male gender (OR: 3.49; 95%CI 1.20 to 10.16, p<0.05), triglycerides (OR: 9.19; 95% CI 2.29 to 36.95, p<0.01), blood viscosity (OR: 1.35; 95% CI 1.01 to 1.81, p<0.05) and body mass index (OR: 1.37; 95% CI 1.14 to 1.64, p<0.01) were independent risks factors for relative hypertension in SCA. No association was found between the BP status and the positive history of painful vaso-occlusive crisis or acute chest syndrome. An association between triglycerides level and the occurrence of these two major acute complications was detected. Our study suggests that male gender, increased triglycerides level, BMI and blood viscosity could increase the risk for developing relative hypertension in SCA. In addition, our results support a role of moderately elevated triglycerides in the pathophysiology of vaso-occlusive events.

## Introduction

Patients with sickle cell anemia (SCA) have lower systolic, diastolic and mean blood pressure (BP) compared with age- and sex-matched controls [1], [2], [3], [4]. The prevalence of hypertension (HTN; BP≥140/90 mmHg) is lower in SCA than in the general population [1], [2], [4], [5]. At that time, no specific recommendations have been proposed by the United States Preventive Services Task Force (USPSTF) regarding the defining criteria (and management) of HTN in SCA. This is a major concern since increased systolic BP (even if lower than 140 mmHg) has been associated with higher risk of stroke [4], [6], [7] and mortality [3] in SCA. However, recently, Gordeuk et al [5] showed that relative HTN (rHTN), defined as BP≥120/70 mmHg in their study, and that HTN (BP≥140/90), considerably increased the risk for pulmonary hypertension and renal dysfunction.

According to the Poiseuille law, blood viscosity is a key component of vascular resistance and mean BP. Several studies, in non-SCA population, reported an association between increased blood viscosity and HTN [8], [9]. Although blood viscosity is usually lower in SCA population compared to the general population [10], elevated blood viscosity increases the risk for vaso-occlusive events in SCA [11], [12], [13]. But, surprisingly, the association of BP and blood viscosity has not been studied so far in SCA and it remains unknown if rHTN, as defined by Gordeuk et al [5] or HTN, could be related to alterations in blood viscosity.

Moreover, a recent growing interest has been devoted to the study of lipid levels in SCA because they might be involved in the pathophysiology of several complications. The lipid profile differs significantly between SCA patients and the general population [14], [15], [16], with SCA patients usually having low levels of total cholesterol and low-density lipoprotein-cholesterol (LDL-C), which is consistent with the virtual absence of atherosclerosis among SCA patients [16]. But, several studies also reported decreased high-density lipoprotein-cholesterol (HDL-C) level in SCA patients [16], [17], which may lead to an increased risk for endothelial dysfunction in this population [16], [18]. This association could be related to the release of oxidized fatty acids during lipolysis, leading to endothelial cell inflammation [19]. Moreover, the atherogenic index of plasma (AIP = log (TG/HDL-C), with TG corresponding to triglycerides level) is increased in SCA and is also a factor of endothelial dysfunction [16]. However, it remains undefined whether this specific lipid profile could favor the development of rHTN or HTN in SCA.

The aim of the present study was to identify biological factors associated with systemic rHTN or HTN (as defined in a previous study [5]) in SCA adult patients to gain insight into the pathophysiology of HTN in this disease. We hypothesized that blood viscosity and lipids profiles are associated with systemic rHTN in adults with SCA.

## Materials and Methods

### Patients

The study took place between May 2010 and December 2011, and included 97 adults with SCA (M/F = 43/54) regularly followed by the Sickle Cell Center at the Academic Hospital of Pointe-à-Pitre (Guadeloupe, French West Indies). All patients were in steady-state condition at the time of the study: no blood transfusions in the previous three months and absence of acute episodes (infection, vaso-occlusive crisis (VOC), acute chest syndrome (ACS), stroke, priapism) at least two months before inclusion into the study. SCA patients under treatment modifying blood rheology were also excluded, except for those on hydroxyurea (HU).

All patients were informed about the purpose and procedures of the study, and gave their written consent. The study was conducted in accordance with the guidelines set by the Declaration of Helsinki and was approved by the Regional Ethics Committee (CPP Sud/Ouest Outre Mer III, Bordeaux, France, registration number: 2010-A00244-35).

### Clinical Parameters

Height (cm) and weight (kg) were measured for all patients. Body mass index was calculated (BMI = weight/height^2^ (Kg/m^2^). Systolic and diastolic BPs were measured as recommended [20]. BP values <120/70 mmHg were considered as normal (NORM group; n = 54). Patients with BP values ≥120/70 mmHg and <140/90 mmHg were considered as having rHTN (n = 38), and those with BP levels ≥140/90 mmHg had HTN (n = 5) [5]. Because of the limited number of patients in the latter group, the two groups with either rHTN or HTN were pooled to create a single group (HTN group; n = 38+5 = 43). Pulse pressure (PP) was calculated for the two groups as the difference between systolic and diastolic BP. Heart rate (HR) and oxygen saturation (SpO2) were measured for each patient. Charts were retrospectively reviewed by two physicians to record all ACS and VOC episodes, as defined in a previous study [11], within the previous year of blood sampling.

### Genetic Parameters

SCA diagnosis was made by isoelectrofocusing (Multiphor II™ System, GE HEALTH CARE, Buck, UK), citrate agar electrophoresis, and cation-exchange high performance liquid chromatography (VARIANT™, Bio-Rad Laboratories, Hercules, CA, USA), and was confirmed by DNA studies [21]. Polymerase Chain Reaction (Gap-PCR) was used to detect 6 common alpha-thalassemia deletions [22], [23].

### Biological Parameters

Blood samples were drawn after a 12-hrs overnight fasting, between 8∶00 a.m. and 10∶00 a.m., and were immediately used for analyses. Measurements of serum lipids levels (total cholesterol, LDL-C, HDL-C and triglycerides (TG)) and hemolytic markers (bilirubine, BIL; lactate dehydrogenase, LDH; aspartate aminotransferase, AST) were performed using standard biochemistry, and AIP was calculated. TG level below 1.69 mM is currently considered as normal. A value of AIP greater than 0.21 suggests increased cardiovascular risk [24], [25], [26]. A principal component analysis was used to derive a hemolytic component from the 4 hemolytic markers measured (i.e. BIL, LDH, AST and reticulocytes expressed in percentage). This standard statistical data reduction approach uses conventional clinical measurements to explain the maximum-shared variance among these indirect measures of hemolysis [27]. The hemolytic component has recently been demonstrated to reflect intravascular hemolysis assessed by measurements of the cell-free plasma hemoglobin [27].

Hemoglobin concentration (Hb), hematocrit (Hct), mean corpuscular volume (MCV), mean corpuscular hemoglobin (MCH), reticulocytes (RET), red blood cell (RBC), platelet (PLT) and white blood cell (WBC) counts were determined using hematology analyzer (Max M-Retic, Coulter, USA).

Blood viscosity was measured after complete oxygenation of the blood, at native Hct, at 25°C and at a shear rate of 225 s*^−^* ^1^ using a cone/plate viscometer (Brookfield DVII+ with CPE40 spindle, Brookfield Engineering Labs, Natick, MA) [28].

### Statistical Analysis

Results are presented as means *±* SD. Unpaired Student’s t-test and chi-square test were used for continuous covariates and for categorical covariates, respectively, to compare biological parameters between the different groups. Pearson test was used for correlations. Principal component analysis was used to derive the hemolytic component.

To identify risk factors associated with HTN in SCA patients, we used a binary (i.e., presence or absence of HTN) multivariate logistic model. Variables of interest at p<0.10 by univariate analysis were included as covariates in the multivariate regression models. Significance level was defined as p*<*0.05. Analyses were conducted using SPSS (v. 20, IBM SPSS Statistics, Chicago, IL).

## Results

### General Characteristics in NORM and HTN Groups

Table 1 shows the general characteristics of the two groups where no difference was observed for age, SpO2, HR, percentages of HbS and HbF, and frequency of patients receiving hydroxyurea treatment. In addition, the proportion of patients with a positive history of VOC or ACS was not different between the two groups. HTN group had higher PP, weight and BMI, compared to the NORM group (p<0.01). Although the difference did not reach statistical significance, the gender distribution tended to differ between the two groups (p<0.10).

**Table 1:** General characteristics of the SCA patients classified according to hypertension.

	NORM	HTN
Sex (M/F)	20/34	23/20
Age (years)	33.9±13.1	35.4±12.7
Height (cm)	169±8	171±10
Weight (kg)	58.7±7.4	66.1±11.1**
Body mass index (kg/m^2^)	20.5±2.5	22.7±3.7**
HbS (%)	84.4±5.8	83.1±5.6
HbF (%)	7.9±5.8	9.1±6.0
HU (%)	24.0	14.3
SpO2 (%)	96.6±2.7	96.6±3.0
PP (mmHg)	44.3±6.7	49.2±10.2**
HR (bpm)	76±10	76±12
Positive history of VOC (%)	13.0	16.3
Positive history of ACS (%)	9.3	9.3

Means ± SD. NORM group, patients with blood pressure values below 120/70 mmHg; HTN group, patients with BP values >120/70 mmHg; HU, hydroxyurea; SpO2, oxygen saturation; PP, pulse pressure; HR, hear rate; VOC, vaso-occlusive crisis; ACS, acute chest syndrome. Significant difference (**p<0.01).

### Hematological Parameters and Hemolytic Profile in NORM and HTN Groups

Table 2 shows the results of the hematological and hemolytic parameters. The two groups were not different regarding alpha-thalassemia frequency, WBC and PLT counts, MCV and MCHC. In contrast, RBC count, Hct and Hb levels were greater in the HTN group in comparison with the NORM group (p<0.01). Except for a trend toward lower bilirubin concentration in the HTN group compared to the NORM group (p<0.1), the other markers were not different between the two groups. However, the hemolytic component derived from the 4 hemolytic markers was lower in the HTN group than in the NORM group (p<0.05). The hemolytic component had a mean of 0 (SD = 1.0) and predicted 49.2% of the variation among all four variables (Eigenvalue = 1.97).

**Table 2:** Hematological parameters and hemolytic profile in SCA patients according to hypertension.

	NORM	HTN
alpha-thalassemia (%)	44.4	41.9
RBC (10^12^/L)	2.7±0.5	3.0±0.7**
WBC (10^9^/L)	9.4±2.4	10.2±2.7
PLT (10^9^/L)	384.8±118.8	412.2±140.6
Hb (g/dL)	8.1±1.2	8.8±1.3**
Hct (%)	22.7±3.3	24.7±3.8**
MCV (fl)	84.6±9.9	84.3±10.1
MCHC (pg)	30.3±3.8	30.1±3.8
RET (%)	217.8±74.8	233.1±73.7
BIL mM	61.2±39.1	56.2±45.9
LDH (UI/L)	510.5±148.3	472.1±157.2
AST (UI/L)	40.7±13.7	36.0±13.0
Hemolytic component (relative unit)	0.20±0.99	−0.24±0.97*

Means ± SD. NORM group, patients with blood pressure values below 120/70 mmHg; HTN group, patients with BP values >120/70 mmHg; RBC, red blood cell count; WBC, white blood cell count; PLT, platelet count; Hb, hemoglobin; Hct, hematocrit; MCV, mean cell volume; MCHC, mean corpuscular hemoglobin concentration; RET, reticulocytes; BIL, bilirubin; LDH, lactate dehydrogenase; AST, aspartate aminotransferase. Significant difference (*p<0.05; **p<0.01).

### Lipids Profile and Blood Viscosity in NORM and HTN Groups

Total cholesterol, HDL-C and LDL-C did not differ between the two groups (Table 3). In contrast, TG levels (p<0.01) and blood viscosity (p<0.05) were greater in the HTN group than in the NORM group. AIP was not significantly different between the two groups (p<0.1) but a significant greater proportion of patients with AIP>0.21, which may suggest higher cardiovascular risk [24], [25], [26], was detected in the HTN group (14.6%) than in the NORM group (7.3%; p<0.05). AIP correlated with HbF (r = −0.26; p<0.05) and PP (r = 0.25; p<0.05).

**Table 3:** Lipid profile and blood viscosity in SCA patients classified according to hypertension.

	NORM	HTN
Total cholesterol (mM)	3.1±0.8	3.2±0.7
HDL-C (mM)	1.0±0.3	0.9±0.1
LDL-C (mM)	1.7±0.6	1.8±0.5
TG (mM)	1.0±0.3	1.2±0.6**
AIP	0.01±0.21	0.10±0.24
Blood viscosity (mPa/s)	5.8±0.8	6.3±1.3*

Means ± SD. NORM group, patients with blood pressure values below 120/70 mmHg; HTN group, patients with BP values >120/70 mmHg; HDL-C, high-density lipoprotein-cholesterol; LDL-C, low-density lipoprotein-cholesterol; TG, triglycerides; AIP, atherogenic index of plasma. Significant difference (*p<0.05; **p<0.01).

At that time, no large study has identified lipids cut-off values above which SCA patients might be at risk for cardiovascular events. The American Heart Association defined cut-off values in the general population with TG level below 1.69 mM being normal. Accordingly to this classification, eighty-five patients had normal TG level, eleven patients had TG level between 1.70 and 2.25 mM (i.e. borderline-high concentration) and one had TG level above 2.25 mM (i.e. high concentration). The proportion of patients with a positive history of VOC or ACS within the previous year was compared between patients with normal TG and those with borderline/high TG. Compared to the group with normal TG level, the group with borderline/high TG level exhibited a greater proportion of patients with a positive history of VOC (33.3 vs 11.9%, p<0.05) and ACS (25.0 vs 7.1%, p<0.05), respectively.

### Multivariate Analysis

A binary multivariate logistic model was used to identify factors associated with HTN in SCA patients and included gender, BMI, TG and blood viscosity. Hb, Hct, hemolytic component or RBC counts were not included in the model to avoid co-linearity effects with blood viscosity. BMI and TG levels were not significantly correlated. PP was not included in the model because it contains some information on the absolute BP level [29], [30]. The overall model was statistically significant (chi-square = 32.90; df = 4; p<0.001) and each of the four parameters included in the model were significantly associated with HTN: male gender (OR: 3.49; 95%CI 1.20 to 10.16, p<0.05), TG (OR: 9.19; 95% CI 2.29 to 36.95, p<0.01), blood viscosity (OR: 1.35; 95% CI 1.01 to 1.81, p<0.05) and BMI (OR: 1.37; 95% CI 1.14 to 1.64, p<0.01). Because blood viscosity could only be modestly affected by the hemolytic rate [31], a second model was tested and included the previous four covariates plus the hemolytic component. This second model was still significant (chi-square = 32.53; df = 5; p<0.001) and retained the same covariates as in the previous model: the hemolytic component was not significant. Finally, although the frequency of HU treated patients between the two groups were not different, we tested a third binary multivariate logistic model including the four co-variates of the first model plus the frequency of patients receiving HU treatment since HU therapy may modulate hematology and blood rheology. We obtained the same results than with the model without HU: i.e., 1) the model was significant (chi-square = 33.679; df = 5; p<0.001) and 2) male gender (OR: 4.20; 95%CI 1.32 to 13.31, p<0.05), TG (OR: 6.89; 95% CI 1.58 to 30.11, p<0.01), blood viscosity (OR: 1.57; 95% CI 1.02 to 2.73, p<0.05) and BMI (OR: 1.48; 95% CI 1.19 to 1.85, p<0.01) were still significantly associated with HTN. In contrast, HU treatment was not significantly associated with HTN (OR: 1.37; 95% CI 0.35 to 5.44).

## Discussion

The present study demonstrates for the first time that 1) male gender, BMI, TG level and blood viscosity are independently associated with relative HTN in SCA; 2) relative HTN is not associated with greater VOC or ACS occurrence; 3) borderline/high TG level is associated with greater VOC or ACS occurrence.

The impact of atherogenic indices and lipoproteins in arterial wall thickness and stiffness has been previously evaluated in children and adolescents not affected by SCA and it has been shown that elevated total cholesterol, TG, LDL-C and low HDL-C levels predict subclinical atherosclerosis in adulthood [32], [33]. However, the lipid profile of SCA population is unique in that these patients exhibit lower LDL-C and HDL-C levels than the general population but greater TG level and AIP [16]. Although sharing common mechanisms with atherosclerosis, SCA vasculopathy clearly differs in that cholesterol accumulation in arterial wall and atheromas have not been reported [16].

Recently, associations were reported between increased TG level or AIP and endothelial dysfunction in SCA patients [16], [18]. These findings suggest that lipids, and more particularly TG level, could play a role in the pathophysiology of several SCA-related complications. High TG level, associated to low HDL-C level, has been demonstrated to have pro-atherogenic, pro-thrombotic and pro-oxidant effects in patients with type 2 diabetes or metabolic syndrome [34]. However, until now, this pathophysiological aspect of SCA has been poorly studied. Our results demonstrated an association between increased TG level and relative HTN or HTN. In addition, the proportion of SCA patients having high AIP was two-fold greater in the HTN group compared to the NORM group. Our findings in SCA adults are in agreement with a recent study conducted in obese and non-obese children showing that increased AIP was associated with pre-hypertension in this population [35]. High TG level and AIP have been both identified in several cardiovascular and autoimmune inflammatory diseases [24], [36], [37], [38]. Although the contribution of TG in the development of cardiac and vascular diseases has been challenged [39], several findings suggest that increased TG level may cause inflammation [19], [40] and oxidative stress in non-SCA population [19], hence modulating vascular function. We did not investigate vascular function in the present study. It has been shown that carotid-femoral or carotid-radial pulse wave velocity is usually lower in SCA patients than in healthy subjects demonstrating that arterial stiffness is reduced in the SCA population [41]. Nevertheless, increased PP in the HTN group could reflect greater arterial stiffness in this subgroup of SCA patients [30], [42] while the positive correlation detected between AIP and PP supports a possible role of lipids in arterial stiffening in the HTN group.

Because of the low Hct and Hb levels, blood viscosity in SCA patients is usually lower than in the general population [10]. However, compared to healthy subjects, the vascular function of SCA patients is altered [43] suggesting that further impairment in blood rheology could be deleterious for the vascular system [44]. In the present study, blood viscosity was increased in the HTN group compared to the NORM group, and remained independently associated with HTN in the multivariate regression model. The increased blood viscosity in the HTN group was probably the consequence of the lower hemolytic rate, which leads to higher Hb and Hct levels. HU treatment and alpha-thalassemia are known to modulate hemolytic rate and the level of anemia, but the percentage of patients with alpha-thalassemia or receiving HU was not different between the two groups [45], [46]. The third binary multivariate logistic model confirmed that HU treatment was not a confounding factor in this study. Whatever the reasons of the increased Hb and Hct levels in the HTN group, the greater blood viscosity may increase vascular resistance and BP [2]. Recently, we reported a positive association between systemic vascular resistance and blood viscosity in children with SCA suggesting that vascular function in this disease is unable to cope with an increase of blood viscosity [44]. We also tested whether the increased TG level could explain the increased blood viscosity in the HTN group but no association between the two parameters was detected (r = −0.15; p = 0.15). Indeed, both increased TG levels and blood viscosity could be at the origin of relative HTN in SCA.

As a consequence of the increased resting energy expenditure caused by the increased erythropoietic and cardiac activities [47], SCA patients are usually characterized by lower BMI values compared to ethnic-matched population. Indeed, in our study, the mean BMI value of the two groups was lower (<25 kg/m^2^) than the limit defining overweight. Nevertheless, we reported an independent association between increased BMI and relative HTN, which confirms previous findings in SCA [3], [48]. Pegelow et al [3] also demonstrated that BP values were higher in male than in females, which is consistent with our findings showing an independent association between male gender and HTN in the multivariate model. Woods et al [49] previously showed that measurement of BMI only, in SCA, was not informative enough to screen for adiposity and obesity. The authors analyzed the body composition of SCA patients with normal mean BMI (22.6 kg/m^2^) and showed a 32.6% fat composition, indicating high levels of adiposity. Since fat accumulation and adipocytes secretion are responsible for many hormonal changes playing a role in the development of vascular dysfunction and HTN in the general population [50], [51], we suggest that it could be the case in SCA patients too, even if BMI values are normal. Further studies are needed to better explore the relationship between BMI, hormonal status and relative HTN in SCA.

Rodgers et al [4] previously suggested that relative HTN and acute vaso-occlusive manifestations could be associated but the authors did not address this issue. Our results do not support this latter hypothesis. However, we observed that SCA patients with borderline/high TG level suffered more frequent VOC or ACS events in the previous year compared to those with normal TG level. The underlying mechanism is unknown at this time but the adverse effects of elevated TG on vascular function and inflammation [16] could explain why increased TG level is associated with VOC and ACS. The pathophysiology of VOC is extremely complex with several interacting factors such as: blood rheology, inflammation, vascular adhesion process, oxidative stress, and now, lipid profile [52]. Further studies are clearly warranted to investigate whether the lipid profile could specifically impact the macro- and microcirculation of SCA patients and ultimately increase the risks for vaso-occlusive events.

Our report clearly suggests that male gender, increased TG level, BMI and blood viscosity could increase the risk for developing relative HTN or HTN. In addition, for the first time, our results support a potential role of moderately elevated TG in the pathophysiology of vaso-occlusive events, thus providing further evidence of a role of lipids in the SCA pathophysiology. Further studies are needed to evaluate the longitudinal associations between HTN, lipid profile and the occurrence of acute and chronic complications in SCA patients. In addition, nutritional aspects have not been investigated in our study, and vitamin D deficiency has been recently reported to participate in the development of hypertension [53]. Further studies are needed to address this issue.
